# An Emerging Target in the Battle against Osteoarthritis: Macrophage Polarization

**DOI:** 10.3390/ijms21228513

**Published:** 2020-11-12

**Authors:** Yulong Sun, Zhuo Zuo, Yuanyuan Kuang

**Affiliations:** Key Laboratory for Space Biosciences & Biotechnology, Institute of Special Environmental Biophysics, School of Life Sciences, Northwestern Polytechnical University, Xi’an 710072, China; zuozhuo@mail.nwpu.edu.cn (Z.Z.); kuangyuanyuan@mail.nwpu.edu.cn (Y.K.)

**Keywords:** macrophages, polarization, osteoarthritis, physical stimuli, chemical compounds, biological molecules

## Abstract

Osteoarthritis (OA) is one of the most prevalent chronic joint diseases worldwide, which causes a series of problems, such as joint pain, muscle atrophy, and joint deformities. Benefiting from some advances in the clinical treatment of OA, the quality of life of OA patients has been improved. However, the clinical need for more effective treatments for OA is still very urgent. Increasing findings show that macrophages are a critical breakthrough in OA therapy. Stimulated by different factors, macrophages are differentiated into two phenotypes: the pro-inflammatory M1 type and anti-inflammatory M2 type. In this study, various therapeutic reagents for macrophage-dependent OA treatment are summarized, including physical stimuli, chemical compounds, and biological molecules. Subsequently, the mechanisms of action of various approaches to modulating macrophages are discussed, and the signaling pathways underlying these treatments are interpreted. The NF-κB signaling pathway plays a vital role in the occurrence and development of macrophage-mediated OA, as NF-κB signaling pathway agonists promote the occurrence of OA, whereas NF-κB inhibitors ameliorate OA. Besides, several signaling pathways are also involved in the process of OA, including the JNK, Akt, MAPK, STAT6, Wnt/β-catenin, and mTOR pathways. In summary, macrophage polarization is a critical node in regulating the inflammatory response of OA. Reagents targeting the polarization of macrophages can effectively inhibit inflammation in the joints, which finally relieves OA symptoms. Our work lays the foundation for the development of macrophage-targeted therapeutic molecules and helps to elucidate the role of macrophages in OA.

## 1. Introduction

Osteoarthritis (OA) is a common degenerative joint disease that causes synovial joint pain and dysfunction [1]. According to the latest forecast, OA will become the leading cause of disability among the global population by 2030 [2]. The hallmark symptoms of OA are joint swelling, joint pain, bone hyperplasia, and limited joint mobility [3,4]. To date, the treatments for OA include medicinal and non-medicinal therapies (such as weight loss and exercise) [5]. However, the pathogenesis of OA is still unclear. It is generally believed that the following factors are likely to trigger OA: gender, obesity, aging, and joint trauma [6,7,8]. Although treatment for OA has been continuously improved in recent years, the demand for more effective clinical therapies remains strong. Therefore, the development of new OA approaches is an urgent task to be undertaken.

Macrophages are essential effector cells of the immune system that participate in a series of biological processes, including immunity, inflammatory responses, and homeostasis [9]. Derived from monocytes, macrophages have substantial heterogeneity and plasticity, and adapt to various microenvironmental stimuli [10,11]. Depending on the stimuli, macrophages are polarized into different phenotypes: M1 and M2.

Macrophage polarization is involved in the pathophysiological processes of many diseases, such as metabolic diseases, pathogen infections, asthma, tumors, and OA. Emerging reports suggest that polarized macrophages are a promising target for the treatment of OA [12], as inhibiting M1 macrophages or promoting M2 polarization can effectively alleviate OA symptoms. Here, we summarize the macrophage polarization-related approaches that have potential therapeutic effects on OA, and explore their mechanisms of action. Our work provides useful clues for the development of macrophage polarization-related therapeutic approaches for OA.

## 2. The Development Stage of Osteoarthritis (OA)

Osteoarthritis is a common chronic disease in aging and obese people that is characterized by the degeneration of articular cartilage [13,14]. OA usually affects human knees, hip joints, and the distal ends of the fingers. With suffering from pain and joint dysfunction, the life quality of OA patients is significantly deteriorated. Although the therapeutic approaches for OA have been continuously improved in recent years, there is still an urgent need for more effective disease-modifying osteoarthritis drugs (DMOADs). At present, the therapeutic measures for OA are divided into two steps: reducing pain and restoring joint function. Clinically, pain relief is the primary target for OA treatment. After the pain is controlled, restoring the joint function of the patient is often accomplished through joint replacement surgery, which leads to huge medical expenses.

Based on the evidence from pathology, OA is divided into early and late stages.

The early stage of OA is as follows:

(a) The activity of osteoclasts in the joints increases, which disrupts the balance between bone formation and bone resorption, and eventually makes the thickness of subchondral bone significantly thinner than normal [15].
(b) The depletion of phagocytic synovial lining cells causes a decrease in the influx of polymorphonuclear neutrophils, which inhibits proteoglycan degradation. Overall, the above changes lead to the death of articular cartilage cells (collagen-induced arthritis (CIA) model). Besides, the reduction of synovial macrophages attenuates the formation of joint osteophytes (in the OA mouse model) [16].
(c) Elevated cholesterol promotes the formation of ectopic bone (CIA model) [17].

The late stage of OA is as follows:

(a) Cartilage is mostly degraded, accompanied by severe pain. At the mechanism level, pain is mainly caused by the following phenomenon: the synovium in the joints of advanced OA patients is infiltrated by macrophages. Subsequently, the activated macrophages release pro-inflammatory cytokines, causing chronic pain and inflammation. From a diagnostic point of view, the levels of three pro-inflammatory mediators in the synovial tissue are significantly up-regulated, which could be considered as a diagnostic marker for OA. The three types of pro-inflammatory mediators are (1) interleukin-1b (IL-1β), (2) IL-6, and (3) nerve growth factor (NGF). Therefore, these indicators may be promising clinical markers for monitoring the progression of OA [18].
(b) The production of IRF5 (interferon regulatory factor 5, IRF5) in the synovial macrophages of OA patients is markedly enhanced, indicating that IRF5 is positively correlated with the severity of OA. Additionally, on the synovial macrophages of OA patients, the IRF5 level in Stage 4 OA patients is significantly higher than that in Stage 2 and Stage 3 [19].
(c) The subchondral plate of OA patients becomes significantly thicker, and the thickness of the unmineralized articular cartilage is significantly thinner than average [15].

## 3. Macrophage Polarization

Macrophage polarization is an effective way to regulate the function of macrophages. As a class of cells in a dynamic equilibrium state, macrophages respond to stimuli in their microenvironment by changing their phenotype and function [20]. According to their differences in phenotype and secreted cytokines, macrophages are classified into two types: the M1 type and M2 type.

M1 macrophages, also called classically activated macrophages, mainly exert pro-inflammatory effects. M1 macrophages are usually activated by toll-like receptors (TLR), which are initially stimulated by interferon-γ, LPS, and TNF-α factors. As for the pro-inflammatory mechanism of M1 macrophages, M1 macrophages firstly secrete a series of cytokines, including IL-1, IL-6, IL-12, IL-23, and TNF-α. Next, the M1 macrophages trigger various immune responses, including killing pathogens and initiating inflammation. Finally, a full-scale inflammatory response breaks out. It is worth noting that an M1 macrophage-provoked excessive inflammatory response usually causes damage to normal tissues, which is reflected as joint pain in OA patients.

M2 macrophages (also referred to as selectively activated macrophages) mostly exhibit anti-inflammatory effects in the immune response. Usually, macrophages are transformed into M2 macrophages by IL-4 and IL-13. Regarding the anti-inflammatory mechanism of M2 macrophages, M2 macrophages secrete large amounts of IL-10, triggering anti-inflammatory and immunosuppressive responses, which finally inhibit inflammation and promote damaged tissue repair. According to different activation methods, M2 macrophages are further classified into three subtypes: M2a, M2b, and M2c [21,22,23].

In recent years, much progress has been made regarding the mechanism of macrophage polarization. In terms of classification, macrophages not only can be classified into two polarization states (namely, M1 and M2), but also are regarded as a large cell family with a broad and plastic phenotype. When being considered as a family of large cells, macrophages perform a variety of physiological functions such as resisting pathogen invasion, maintaining homeostasis, and regulating the inflammatory state of the local microenvironment [11].

According to the latest findings, macrophages are classified into the following categories: (a) tumor-associated macrophages (TAM): a subtype of macrophages related to cancer; (b) CD169^+^ macrophages: a subtype found in lymphoid organs that anticipates immune tolerance and antigen presentation; (c) T-cell receptor-positive (TCR^+^) macrophages: a recently discovered macrophage subtype. Collectively, emerging data show that the above macrophage subtypes play unique roles in the entire macrophage functional process [24].

## 4. Macrophages Are an Emerging Target for OA Treatment

One useful measure for treating OA is to restore the balance of the immune microenvironment by regulating the macrophages in the joints of OA patients. As is well known, OA is an immune disorder caused by immune microenvironment malfunction. Many factors are capable of affecting the occurrence and development of OA, including physical stimuli, chemical compounds, and biological molecules. Increasing findings show that the above OA treatment approaches modulate inflammation by reprogramming macrophages. Here, we summarize the mechanisms of regulating macrophages to affect OA, which provide clues for an in-depth understanding of the regulatory roles of macrophages in OA (Table A1).

### 4.1. Physical Stimuli

#### Low-Intensity Pulsed Ultrasound (LIPUS)

**Figure 1:**
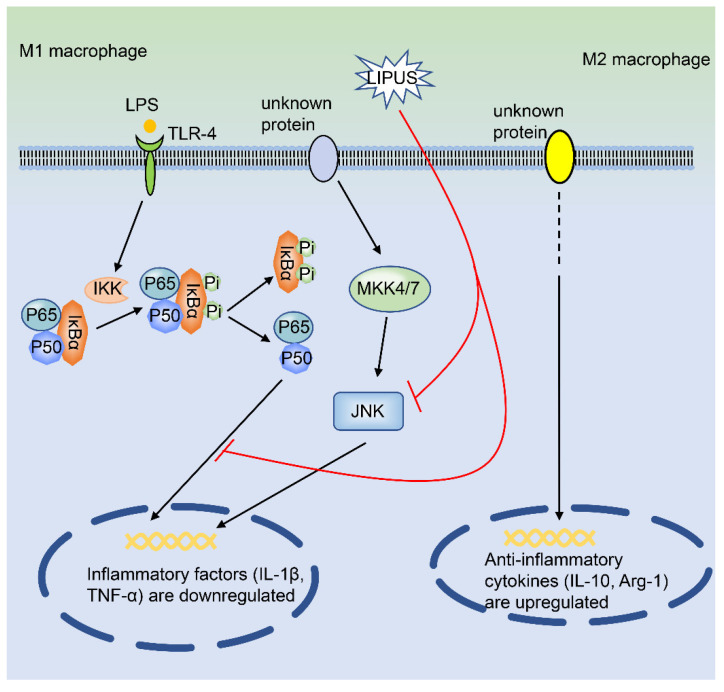
LIPUS regulates the polarization of macrophages. LIPUS significantly inhibits the LPS-induced activation of p-JNK and p-p65 in macrophages. At the same time, LIPUS down-regulates the expression of IL-1β and TNF-α mRNA, whereas it up-regulates the expression of IL-10 and Arg-1, in macrophages. LIPUS, low-intensity pulsed ultrasound; IL-1β, interleukin-1β; IL-10, interleukin-10; Arg-1, arginase-1; TNF-α, tumor necrosis factor-α; JNK, c-Jun N-terminal kinase; NF-κB, nuclear factor kappa-light-chain-enhancer of activated B cells; LPS, lipopolysaccharide.

Low-intensity pulsed ultrasound (LIPUS) is a kind of low-frequency, low-power ultrasound [25]. In recent years, due to its unique characteristics of non-invasiveness and safety, LIPUS has been widely used as a diagnostic and therapeutic tool [25,26]. In a mouse model of destabilization of medial meniscus (DMM) arthritis, Zhang et al. found that LIPUS significantly alleviated the OA symptoms. *At the cellular level*, LIPUS treatment decreases the proportion of M1 macrophages and increases the M2 macrophages in the joint synovium. Besides, after being exposed to LIPUS, the thickness of the synovial membrane of the mouse knee joint became thinner than that in the control group. *In in vivo experiments*, the intra-articular injection of LIPUS makes the synovial membrane of the mouse knee joint thinner than that of the control group. *At the signaling pathway level*, LIPUS inhibits the mRNA expression of M1 macrophage-related genes in THP-1 and RAW264.7 cells but promotes the gene expression of M2 macrophage-related genes in the LPS-induced macrophage inflammation model. Hence, LIPUS attenuates inflammation signaling pathways in different macrophage models, as it remarkably suppresses LPS-mediated signaling pathways in THP-1 cells (p-JNK and p-NF-κB p65) [27] (Figure 1). In summary, LIPUS is expected to become a potential therapeutic approach for OA therapy.

### 4.2. Chemical Compounds

#### 4.2.1. Kinsenoside

Kinsenoside (Kin) is a steroid immunosuppressant that is derived from the traditional Chinese herbal medicine *Anoectochilus roxburghii* [28]. At present, Kin is widely used in various clinical diseases, including tumors, osteoporosis, hyperlipidemia, and hyperglycemia [28,29].

**Figure 2:**
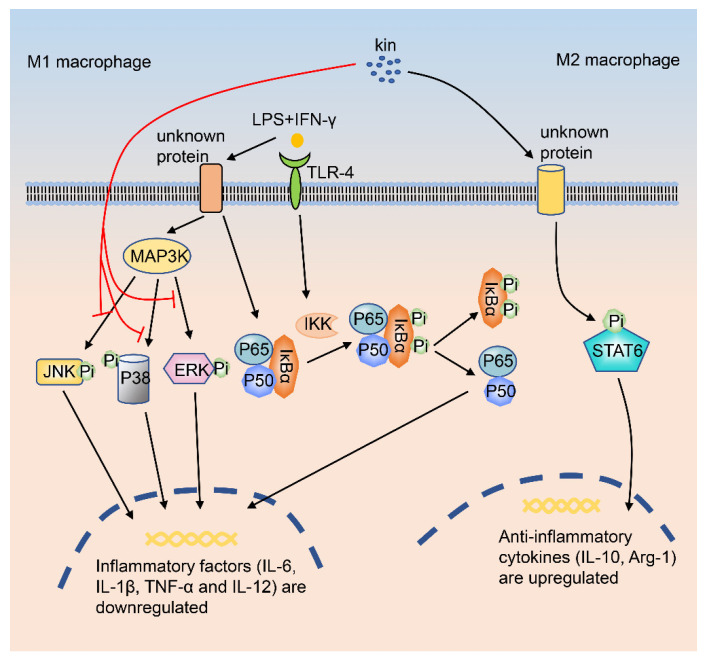
Kin regulates macrophage polarization through MAPK, NF-κB, and STAT6 signaling pathways. When macrophages are stimulated by LPS+IFN-γ, the major components of the NF-κB signaling pathway (IKK, IκBα, and p65) are phosphorylated sequentially. Kin inhibits the activation of a series of signaling pathways in macrophages, including the p-JNK, p-ERK, p-P38, and p-p65 signaling pathways. Finally, the expression of M1-related genes (IL-6, IL-1β, TNF-α, and IL-12) in macrophages is down-regulated by Kin. Kin, kinsenoside; MAPK, mitogen-activated protein kinase; NF-κB, nuclear factor-κ-gene binding; STAT6, signal transducer and activator of transcription 6; LPS, lipopolysaccharide; IFN-γ, interferon-γ; JNK, c-Jun N-terminal kinase; ERK, extracellular regulated protein kinase.

Recently, the role of Kin in regulating macrophages has been reported. Zhou et al. investigated the effects of different concentrations of Kin on macrophage repolarization and inflammation, and found that Kin reprograms M1 macrophages into M2 macrophages. *In in vivo experiments*, by using an OA mouse model (anterior cruciate ligament transection, ACLT), Kin reduces the infiltration of M1 macrophages in the joints and promotes the infiltration of M2 macrophages in the synovium. In addition, Kin inhibits the destruction of subchondral bone and reduces the damage to the articular cartilage. *At the cellular level*, Kin attenuates chondrocyte damage induced by the co-culture of macrophages in CM (conditioned medium) and IL-β. *At the signaling pathway level*, Kin suppresses the phosphorylation of IκBα, p-JNK, p-ERK, and p-P38 in macrophages (Figure 2). Besides, Kin down-regulates the production of inflammatory cytokines, which finally promotes the transformation of M1 macrophages into M2 macrophages. Besides, Kin also enhances the function of M2 macrophages via enhancing M2 macrophage markers [15].

#### 4.2.2. Quercetin

Quercetin is a kind of flavonoid that is widely distributed in fruits and vegetables [30]. Quercetin has powerful anti-inflammatory, antioxidant, and anti-allergic effects [30,31,32]. Because quercetin directly regulates the polarization of synovial macrophages in the joints of OA patients, it is considered to be a potential therapeutic molecule for OA treatment.

Quercetin promotes the transition of synovial macrophages to M2 macrophages, thereby attenuating the symptoms of OA.

*In in vitro experiments*, quercetin directly down-regulates the expression of inducible nitric oxide synthase (iNOS), cyclooxygenase 2 (COX2), and matrix metalloproteinase-13 (MMP-13) in IL-1β-treated rat chondrocytes [33]. In a co-culture system consisting of chondrocytes and macrophages, quercetin protects cartilage from degradation by promoting the formation of M2 macrophages.

**Figure 3:**
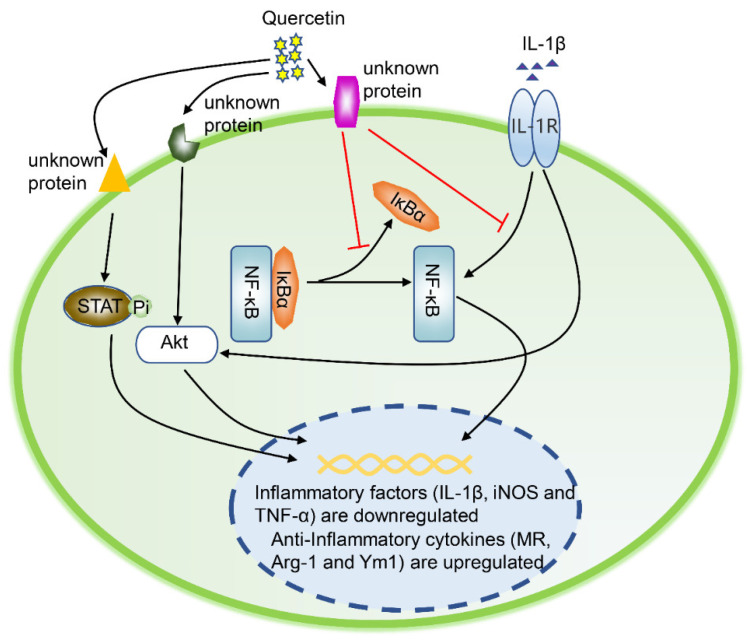
Quercetin regulates the polarization of macrophages. In the inflammatory state caused by IL-1β, a series of signaling pathways in macrophages are activated, including AKT phosphorylation, IκBα degradation, and p65 phosphorylation. In response to quercetin stimuli, STAT6 and Akt are phosphorylated, promoting STAT6’s entry to the nucleus. Subsequently, the expression of M2 macrophage-related genes (MR, Arg-1, and Ym1) is up-regulated. Eventually, the macrophages are polarized into M2. IL-1β, interleukin-1β; AKT, protein kinase B; IκBα, inhibitor of NF-κB; STAT6, signal transducer and activator of transcription 6; MR, mannose receptor; Ym1, chitinase 3-like protein 3; Arg-1, arginase-1.

*In in vivo experiments*, the intra-articular injection of quercetin significantly reduces the level of chondrocyte p-Akt (Figure 3). Besides, quercetin inhibits the NF κB signaling pathway via inhibiting IkBα degradation and p65 phosphorylation, and blocking the transport of p65 from the cytoplasm to the nucleus (Figure 3). Overall, in vivo and in vitro experimental data show that quercetin has a practical anti-apoptotic effect on OA chondrocytes.

*At the signaling pathway level*, quercetin promotes the formation of M2 macrophages by activating the Akt/STAT6 signaling pathway in a time-dependent manner (Figure 3). Specifically, quercetin ameliorates OA symptoms in several manners, including inhibiting the Akt/STAT6 signaling pathway, suppressing inflammatory factor production (iNOS, COX2, MMP-13, and ADAMTS-4), and reversing IL-1β-induced cartilage matrix degradation [34] (Figure 3).

#### 4.2.3. Dexamethasone

Dexamethasone (DXMS) is a synthetic corticosteroid with a variety of pharmacological effects, including anti-inflammatory, anti-rheumatic, anti-toxic, and anti-allergic activities [16]. *In in vitro experiments*, dexamethasone alleviates the symptoms of OA patients by inhibiting the inflammatory response of synovial macrophages. *At the cellular level*, dexamethasone suppresses M1 macrophage activity and promotes the formation of M2 macrophages. *At the gene expression level*, dexamethasone enhances the expression of CD163 (a marker for anti-inflammatory activity [35]) on the surface of synovial macrophages. *In an animal model of post-traumatic OA*, dexamethasone (intra-articularly) protected the cartilage from degradation and suppressed inflammation in the joints [16].

It is worth noting that glucocorticoid drugs (such as dexamethasone) also have obvious negative effects, including causing electrolyte disorders, abnormal blood lipid metabolism, adrenal cortical dysfunction, mental disorders, osteoporosis and even femoral head necrosis [36]. In particular, glucocorticoid-induced osteoporosis is the primary cause of osteoporosis in young people [36]. Therefore, we should be very cautious when using glucocorticoids to treat patients with osteoarthritis.

#### 4.2.4. Pravastatin

Statins are a class of cholesterol synthesis inhibitors that have a wide range of immunomodulatory and anti-inflammatory activities [37]. Pravastatin, an essential member of the statin family, has shown promising activity in the treatment of OA. *At the level of polarized macrophages*, pravastatin promotes the formation of M2 macrophage in several inflammation models [38,39]. Moreover, pravastatin up-regulates the level of the anti-inflammatory marker CD163 on M2 macrophages. In line with this, statins also relieve OA symptoms by regulating macrophage polarization. Furthermore, Utomo et al. report that pravastatin inhibits the secretion of IL-10 by macrophages in the joints [16]. Overall, pravastatin is a potential macrophage-targeted therapeutic reagent for OA therapy.

#### 4.2.5. Rapamycin

Rapamycin is a widely used immunosuppressant that suppresses the activation of T cells by inhibiting the signal transduction of the target of rapamycin (mTOR) [40]. mTOR is a protein kinase that controls the growth, proliferation, and survival of cells [41]. Mounting evidence demonstrates that the mTOR pathway plays a crucial role in macrophage polarization [42,43,44,45,46].

mTOR is a regulator of cell metabolism that contains two different complexes: mTORC1 and mTORC2. Constitutive mTOR complex 1 (mTORC1) activation promotes M1 macrophage formation, but it cannot polarize the macrophages toward the M2 phenotype [16]. Therefore, mice with mTORC1 activation or inhibition in the bone marrow lineage are ideal models for exploring the polarization of macrophages in OA. Zhang et al. show that the activation of mTORC1 in the synovium enhances M1 macrophages and aggravates the symptoms of collagenase-induced osteoarthritis (CIOA). However, the mTORC1-mediated suppression of M2 macrophages protects mice from collagenase-induced cartilage degradation and osteophyte formation [47]. Hence, selective mTOR inhibitors are practical approaches to treating OA with novel mechanisms of action in macrophages.

### 4.3. Biological Molecules

#### 4.3.1. Cells

##### Mesenchymal Stem Cells

Mesenchymal stem cells (MSCs) are a type of pluripotent stem cells that exist in a variety of tissues, including bone marrow, skeletal muscle, periosteum, and trabecular bone [48]. Clinically, MSCs are used to treat various diseases, such as hematological malignancies, cardiovascular diseases, liver cirrhosis, nervous system diseases, and OA [49,50]. By down-regulating the response of immune cells, MSCs promote immune suppression and enhance immune tolerance. Besides, MSCs regulate a variety of innate immune cells, such as macrophages, dendritic cells, natural killer cells, and adaptive immune cells.

MSCs inhibit inflammation by converting macrophages to the M2 phenotype. However, it has not been reported that MSCs directly regulate the synovial macrophage phenotype of OA patients. *In in vitro experiments*, MSCs have blocked the activation of M1 macrophages and induced the polarization of M2 macrophages. Interestingly, M1 macrophages also regulate MSCs in turn, as M1 inhibit the proliferation and viability of MSCs. Finally, at the joints of OA patients, M1 macrophages enhance the immune response and finally accelerate cartilage degradation.

In the joints of OA patients, MSCs firstly migrate to the site of tissue damage. Subsequently, MSCs promote the transition of macrophages into M2 macrophages by secreting a large number of chemokines, cytokines, and growth factors [46]. Finally, M2 macrophages promote the repair of damaged tissues. *In the mouse OA model,* Hamilton et al. show that MSC treatment (intra-articular injection) down-regulates the level of iNOS in macrophages, and finally decreases the formation of M1 macrophages [51]. Collectively, the above findings suggest that MSCs suppress inflammation by converting macrophages into the M2 phenotype, which would make them a promising therapeutic reagent for OA.

##### TissueGene-C

TissueGene-C is a gene therapy method that delivers TGF-β1 to target cells [52]. In recent years, TissueGene-C has shown promising prospects in the treatment of OA. According to a report by Choi and colleagues, TissueGene-C promotes the formation of M2 macrophages in the joints of OA patients and enhances their anti-inflammatory activity, which finally reduces pain and promotes cartilage regeneration. In a monosodium iodoacetate (MIA) rat model of OA, the expression of IL-10 (together with other M2 macrophage markers) was significantly enhanced in the knee joints of the TissueGene-C treatment group. In the rat MIA model, TissueGene-C (intra-articular injection) promotes the formation of M2 macrophages in the knee joint and significantly enhances M2 macrophage-secreted IL-10 [52]. The above data demonstrate that TissueGene-C favors an anti-inflammatory environment in the knee joint.

Encouragingly, the first Phase II clinical trial using TissueGene-C to treat OA shows that TissueGene-C significantly improves a series of symptoms of OA patients, including pain, daily activities, and motor function. Currently, the Phase III clinical trial of TissueGene-C is recruiting OA patients, which makes it possible for TissueGene-C to be the first disease-modifying osteoarthritis drug (DMOAD) [53]. Taken together, by inhibiting M1 macrophages or promoting M2 macrophages, TissueGene-C could be a practical therapeutic approach for OA therapy.

#### 4.3.2. Proteins

##### R-Spondin-2

R-spondin-2 (Rspo2), a protein secreted by M1 macrophages, plays an important role in the development of OA. Zhang et al. demonstrate that Rspo2 aggravates the process of experimental OA, hinting that inhibiting Rspo2 could be a potential approach for OA therapy. Although many reports have confirmed that Rspo2 is an activator of the Wnt/β-catenin signaling pathway [54], the role of Rspo2 in OA has not been fully confirmed yet. During the process of endochondral ossification, Rspo2 activates Wnt/β-catenin signaling and hence promotes the hypertrophy and differentiation of chondrocytes [55]. Additionally, another in vitro study conducted in MC3T3-E1 cells (a pre-osteoblastic cell line) demonstrates that Rspo2 is a positive regulator of bone metabolism [56].

Interestingly, an antibody targeting Rspo2 can effectively suppress the β-catenin signaling pathway in articular chondrocytes and ultimately delays the development of OA in mice. Overall, M1 macrophage-secreted Rspo2 alleviates cartilage degeneration in OA joints and promotes joint soft tissue repair. It is worth noting that the regulation of macrophage polarization by Rspo2 has not been reported yet [47]. Hence, it could be an exciting direction to investigate the effect of Rspo2 on the pathogenesis of OA by targeting the polarization of macrophages.

##### Interferon Regulatory Factor 5

Interferon regulatory factor 5 (IRF5) is a transcription factor encoded by the IRF5 gene, a member of the interferon regulatory factors family [57]. IRF5 plays a vital role in the occurrence and development of many diseases, such as tumors, systemic lupus erythematosus, and OA [57]. In the treatment of OA, IRF5 has shown promising prospects.

*At the cellular level*, (a) IRF5 promotes M1 polarization and enhances the differentiation of Th1 and Th17 cells [58]. (b) In patients with advanced OA, the levels of IRF5, IL-12p35, and IL-12p40 in synovial macrophages were significantly higher than those of patients with early OA [19]. Given that high IL-12 expression is a hallmark of M1-polarized macrophages, synovial macrophages should have strong M1-like characteristics [19].

From a genetic point of view, mice with IRF5 gene knockouts have significantly less inflammation in joints than wild-type mice. Besides, these IRF5-modified mice have lower levels of IL-1β than wild-type mice. Moreover, research by Ni et al. suggests that the expression of IRF5 is positively correlated with the severity of OA, which may contribute to the activation of the M1–Th1 axis. Collectively, IRF5 promotes the formation of M1 macrophages and accelerates the development of OA [19]. Hence, IRF5 inhibitors or nucleic acid reagents might be useful in OA therapy.

##### Pro-Resolving Lipid Mediator

Retinoid D1 (RvD1) is a mediator that promotes the breakdown of lipids, with potent anti-inflammatory and decomposing properties. In particular, RvD1 has significant efficacy in altering the pro-inflammatory behavior of macrophages. A large number of epidemiological data show that obesity is positively correlated with the incidence of OA. Sun et al. show that RvD1 reduces the expression of pro-inflammatory genes, increases the expression of anti-inflammatory genes (inducing the polarization of M2 macrophages), and finally alleviates the development of obesity-related OA [59].

In in vivo experiments, (a) clodronate-loaded liposomes (CL) relieved synovitis in an obese mouse model with post-traumatic osteoarthritis (PTOA). Besides, CL prevent cartilage destruction. (b) RvD1 reduces the levels of pro-inflammatory markers in CD14^+^ human macrophages. In terms of the mechanism of action, RvD1 (intra-articular treatment) significantly reduces the symptoms of mouse knee joint OA, which is accomplished in the following ways: (a) attenuating the infiltration of synovial macrophages, (b) reducing the number of pro-inflammatory macrophages in the synovium, and (c) reversing the synovitis and degradation of cartilage [59].

##### Lumican

Lumican (LUM) is a small, leucine-rich extracellular proteoglycan that exists in many tissues, including skin, muscle, and articular cartilage [60]. Recently, the role of LUM in regulating the polarization of macrophages has been revealed. In LPS-induced M1 and IL-4-mediated M2 macrophage models, co-stimulation with LUM and LPS enhances the activation of M1 macrophages, whereas LUM and IL-4 co-stimulation inhibits the activation of M2 macrophages. *At the level of gene expression*, the mRNA levels of LUM and TLR are up-regulated in cartilage and synovial cells [61,62]. *At the pathophysiological level*, LUM promotes LPS-induced TLR4 activation, which finally leads to extensive cartilage degradation. Besides, the co-stimulation of LUM and LPS also promotes macrophage activation and causes polarization towards the M1 phenotype [63].

Interestingly, LUM alone does not induce any inflammatory response. Very recently, Barreto and colleagues demonstrated that intact LUM in synovial fluid enhances the LPS-induced inflammatory response in a TLR4-dependent manner, indicating that LUM may promote the formation of OA through this pathway [23,64]. *In terms of signaling pathways*, LUM significantly activates the NF-kB signaling pathway in macrophages in OA joints [63]. In summary, LUM exacerbates the process of inflammation by acting on macrophages in OA joints.

##### Bone Morphogenetic Protein 7

Bone morphogenetic protein 7 (BMP-7) is a member of the transforming growth factor-beta (TGF-β) superfamily [65]. Clinically, BMP-7 is mostly used in the research and application of plastic surgery, such as that treating long bone nonunion fractures [66]. Recently, the influence of BMP-7 on OA has attracted increasing attention.

BMP-7 has a biphasic effect on synovial tissue under different conditions, where BMP-7 promotes inflammation on the synovium stimulated by IFNγ+TNFα, and enhances anti-inflammatory activity on OA synovium in the absence of IFNγ+TNFα. Therefore, the anti-inflammatory effect of BMP-7 depends on the cell types in the local tissue. It is worth noting that the mechanism of BMP-7’s pro-inflammatory effect on IFNγ+TNFα-stimulated synovial tissue is still unclear, which may be due to the decrease in the IL-1RA-mediated M1/M2 index. However, in monolayer synovial tissue, the regulation of M1 macrophages by BMP-7 has not been reported. Based on the above findings, BMP-7 may directly regulate other cells rather than macrophages in the local environment of inflammation. Moreover, BMP-7 has a direct therapeutic effect on OA, as the injection of BMP-7 into the articular cavity significantly alleviates OA symptoms in experimental OA models [16]. Collectively, BMP-7 is an appealing macrophage-targeted therapeutic reagent for OA.

##### Squid Type II Collagen

Squid type II collagen (SCII) is a classically recognized essential collagen component in articular cartilage that plays a vital role in the development and maturation of cartilage cells. SCII or SCII-derived composite materials have attracted widespread attention for OA therapy [67]. SCII relieves OA symptoms by promoting the formation of M2-type macrophages. In the joints of OA patients, SCII promotes the transformation of macrophages into the M2 phenotype [46]. In addition, SCII inhibits the pathological apoptosis and hypertrophy of chondrocytes, and finally promotes cartilage repair [68].

The mechanism of SCII in treating OA has already been confirmed in an OA rat model. Specifically, SCII increases the formation of M2 macrophages and chondrogenic factors (TGF-β1 and TGF-β3) in the synovial fluid, and finally inhibits chondrocyte apoptosis and MMP13 production. *At the cellular level*, SCII promotes the transition of macrophages from the M0 to M2 phenotype, thereby exerting an immunomodulatory activity. *At the signaling pathway level*, SCII enhances the phosphorylation of STAT6 (p-STAT6) and transfers p-STAT6 to the nucleus in macrophages [68].

**Figure 4:**
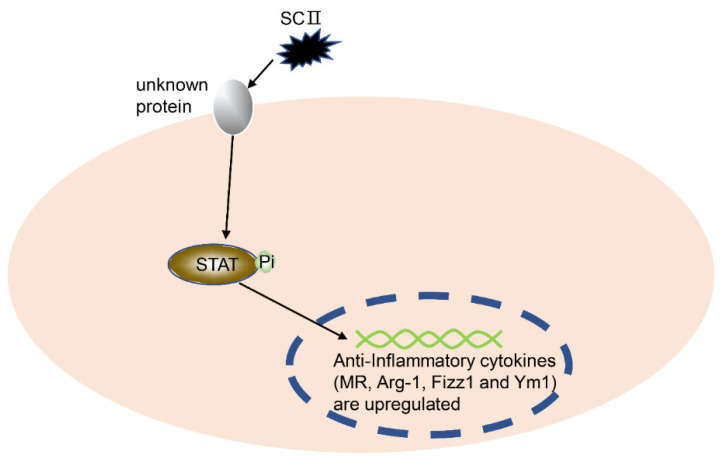
SCII regulates the polarization of macrophages. After SCII treatment of macrophages, the phosphorylation level of STAT6 was significantly increased, and the transfer of p-STAT into the nucleus was promoted. Finally, the macrophages were polarized to M2. SCII, squid type II collagen; STAT6, signal transducer and activator of transcription 6.

Because the activation of STAT6 and nuclear transfer are the critical events for the polarization of M0 macrophages to M2 macrophages [69,70], the SCII-induced phenotypic change in macrophages (from M0 to M2) is hence closely related to the STAT6 signal pathway (Figure 4). In summary, SCII would be a novel cartilage repair biomaterial reagent for OA therapy.

##### Modified ZIF-8 Nanoparticles

Zeolitic imidazolate framework-8 (ZIF-8) is a porous crystalline material formed by the coordination of zinc ions and 2-methylimidazole that has excellent biocompatibility. Therefore, ZIF-8 is considered to be an ideal carrier for controlling the transportation and release of medicine that has excellent potential in biomedicine applications [71,72].

Recently, the potential of ZIF-8 in the treatment of OA has been investigated. Zhou et al. established a model, modified ZIF-8 nanoparticles (ZIF-8 NPs), which regulate the conversion of M1 macrophages to the M2 phenotype and inhibit the activity of synovial M1 macrophages in OA joints [73].

*At the in vivo level*, in a mouse knee joint OA model (anterior cruciate ligament transection, ACLT), NPs (intra-articular and intravenous injection) relieved a series of OA syndromes; for example, (1) the intra-articular injection of NPs (one time/4 d) effectively relieved OA symptoms; (2) the modified NPs inhibited cartilage degeneration and the hypertrophy of chondrocytes induced by macrophage CM [73].

**Figure 5:**
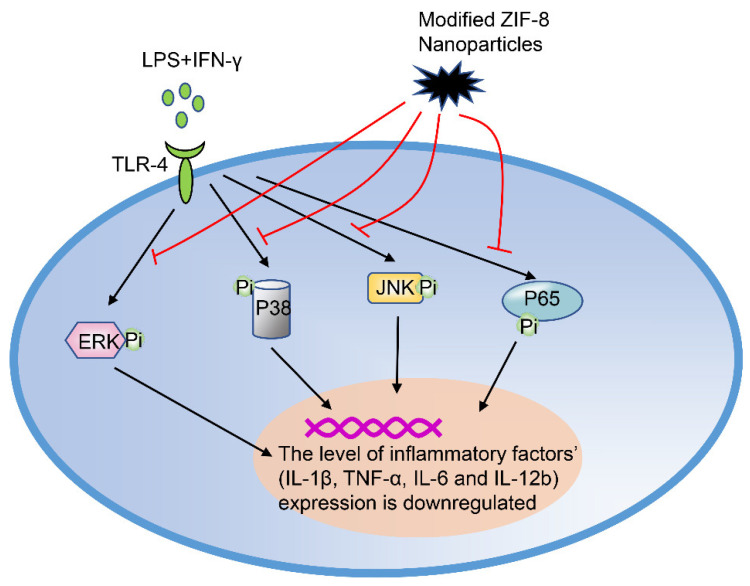
Modified ZIF-8 nanoparticles regulate the polarization of macrophages. In an inflammatory state, inflammatory factors (LPS+IFN-γ) cause the phosphorylation of ERK, JNK, p38, and p65, which subsequently activate the downstream signaling pathways respectively. However, the phosphorylation of these (p-ERK, p-p38, and p-p65) was inhibited by modified ZIF-8 nanoparticles. LPS, lipopolysaccharide; IFN-γ, interferon-γ; ERK, extracellular regulated protein kinase; JNK, c-Jun N-terminal kinase.

*At the signaling pathway level*, reactive oxygen species (ROS) mediate the activation of the MAPK and NF-κB (p65) signaling pathways, which are essential for the polarization of M1 macrophages. The modified ZIP-8 nanoparticles inhibit the expression of p-ERK, p-p38, and p-p65 in macrophages, without affecting the whole p-JNK pathway (Figure 5). Overall, these findings show that the ROS-mediated signaling pathway is involved in the process of regulating macrophages by modified NPs [73].

*At the cellular level*, ZIF-8 NPs directly regulate many aspects of macrophages, including (1) inhibiting inducible NO synthase and thereby reducing NO production; (2) catalyzing the conversion of H_2_O_2_ to produce O_2_ and eliminating NO, thus suppressing hypoxia-inducible factor 1α; (3) in ACLT-induced synovial tissues, decreasing the infiltration of synovial M1 macrophages (CD16/32 positive cells) and promoting the formation of synovial M2-phenotype macrophages. In summary, in the ACLT-induced OA mouse model, the modified NPs protect articular cartilage from degradation and reduce chondrocyte apoptosis [73]. Overall, the ZIF-8 nanoparticles promote the transition of macrophages from a pro-inflammatory M1 phenotype to an anti-inflammatory M2 phenotype, which makes it a promising therapy for OA.

## 5. Conclusions

At present, the unsatisfactory drug treatment effect for osteoarthritis has brought a heavy burden on the global health system. After the articular cartilage is worn to a certain extent, it causes a local immune microenvironment disorder of the joint. This local microenvironment disorder triggers a variety of uncontrolled inflammatory reactions. Once these inflammatory reactions are initiated, it is a promising strategy to focus on restoring the homeostasis of the local immune microenvironment in the joints from the early stage of osteoarthritis. Macrophages are a natural component in the local immune microenvironment of joints, and the polarization of macrophages is the key to defeating OA. In the future, it will be an effective strategy to develop therapeutic measures against OA by targeting the polarization processes of macrophages (polarization towards M2).

Regarding the achievable time frame for the above-mentioned treatment methods, most of the treatment methods are still in the preclinical research stage and will take at least 5–10 years to reach patients with osteoarthritis. Encouragingly, there are also approaches currently in clinical trials (such as TissueGene-C, in Phase III of a clinical trial), which brings hope to osteoarthritis patients.

## Abbreviations

Abbreviation	Full name
ACLT	Anterior cruciate ligament transection
ADAMTS-4	A disintegrin and metalloproteinase with thrombospondin motifs 4
Akt	Protein kinase B
Arg-1	Arginase-1
BMP-7	Bone morphogenetic protein 7
CIA	Collagen-induced arthritis
CIOA	Collagenase-induced osteoarthritis
CL	Clodronate-loaded liposomes
CM	Conditioned medium
COX2	Cyclooxygenase 2
DMOADs	Disease-modifying osteoarthritis drugs
DXMS	Dexamethasone
ERK	Extracellular regulated protein kinase
H_2_O_2_	Hydrogen peroxide
IFN-γ	Interferon-γ
IL-1	Interleukin-1
IL-1β	Interleukin-1β
IL-4	Interleukin-4
IL-6	Interleukin-6
IL-10	Interleukin-10
IL-12	Interleukin-12
IL-13	Interleukin-13
IL-23	Interleukin-23
iNOS	Inducible nitric oxide synthase
IRF5	Interferon regulatory factor 5
IκBα	Inhibitor of NF-κB
JNK	C-Jun N-terminal kinase
Kin	Kinsenoside
LIPUS	Low-intensity pulsed ultrasound
LPS	Lipopolysaccharide
LUM	Lumican
MAPK	Mitogen-activated protein kinase
MIA	Monosodium iodoacetate
MMP-13	Matrix metalloproteinase-13
MR	Mannose receptor
MSCs	Mesenchymal stem cells
mTOR	Mammalian target of rapamycin
NF-κB	Nuclear factor kappa B
NGF	Nerve growth factor
NO	Nitric oxide
O_2_	Oxygen
OA	Osteoarthritis
PTOA	Post-traumatic osteoarthritis
ROS	Reactive oxygen species
Rspo2	R-spondin-2
RvD1	Retinoid D1
SCII	Squid type II collagen
STAT6	Signal transducer and activator of transcription 6
TAM	Tumor-associated macrophages
TCR^+^	T-cell receptor-positive
TGF-β	Transforming growth factor-beta
TGF-β1	Transforming growth factor-beta 1
TGF-β3	Transforming growth factor-beta 3
TLR	Toll-like receptor
TLR4	Toll-like receptor 4
TNF-α	Tumor necrosis factor α
YM1	Chitinase-like protein
ZIF-8	Zeolitic imidazolate framework-8
ZIF-8 NPs	ZIF-8 nanoparticles

## Appendix A

**Table A1:** Macrophage-based therapeutic strategies for OA.

Type		Name	OA Sample—Cells	OA Sample—Animal	Signaling Pathway	Function	Research Stage/Application Schedule	Mechanism	Refs.
**Physical stimuli**		Low-intensity pulsed ultrasound (LIPUS)	THP-1 cells; RAW 264.7; synovial macrophages	Mouse medial meniscus instability (DMM) arthritis	JNK NF-κB	Inhibit the expression of related genes in M1 macrophages and promote the expression of related genes in M2 macrophages	Preclinical stage/NA	LIPUS regulates the polarization of synovial macrophages, thus inhibiting osteoarthritis.	[27]
**Chemical compounds**		Kinsenoside (Kin)	RAW264.7	Anterior cruciate ligament transection (ACLT) mouse model	NF-κB JNK MAPK ERK	Transform M1 macrophages into M2 macrophages; reduce the infiltration of M1 in mouse joints and promote the infiltration of M2 in mouse synovitis	Preclinical stage/NA	Kinsenoside relieves the symptoms of osteoarthritis by inactivating NF-κB/MAPK signaling and promoting macrophage repolarization.	[15]
		Quercetin	RAW264.7	IL-1β-induced rat osteoarthritis model	Akt/NF-κB	Promote the formation of M2 macrophages	Preclinical stage/NA	Quercetin repairs damaged cartilage by promoting the conversion of synovial macrophages into M2 macrophages, and finally alleviates OA symptoms.	[34]
		Dexamethasone (DXMS)	Synovial macrophages	Patients with osteoarthritis	NA	Inhibit the activity of M1 macrophages and promote the formation of M2 macrophages	Preclinical stage/NA	DXMS promotes the conversion of macrophages into M2 macrophages to relieve OA symptoms.	[16]
		Pravastatin	Primary human monocytes	Patients with osteoarthritis	NA	Promote the formation of M2 macrophages	Preclinical stage/NA	DXMS promotes the conversion of macrophages into M2 macrophages to relieve OA symptoms.	[16]
		Rapamycin	Primary human monocytes	Patients with osteoarthritis	mTOR	Enhance the function of M1 macrophages and inhibit the activity of M2 macrophages	Preclinical stage/NA	DXMS promotes the conversion of macrophages into M2 macrophages to relieve OA symptoms.	[16,47]
**Biological molecules**	**Cell**	Mesenchymal stem cell (MSC)	Synovial macrophages of OA patients	In vitro co-culture OA model consisting of patient-matched cartilage and macrophages	NA	Inhibit the activity of M1 macrophages and induce polarization of M2 macrophages	Preclinical stage/NA	MSCs promote the formation of M2 macrophages, thereby relieving OA symptoms	[46,51]
		TissueGene-C	Human joint macrophages	Patients with osteoarthritis	NA	Promote the formation of M2 macrophages	Phase III of a clinical trial/NA	TissueGene-C induces the formation of M2 macrophages, thereby relieving pain in patients with OA.	[52,53]
	**Protein**	R-spondin-2	NA	NA	Wnt/β-catenin	NA	Preclinical stage/NA	The formation of M1 macrophages promoted the secretion of rspo2 in chondrocytes, thereby exacerbating the symptoms of experimental OA.	[47]
		Interferon regulatory factor 5 (IRF5)	Synovial macrophages	Patients with osteoarthritis	NA	Promote the formation of M1 macrophages	Preclinical stage/NA	M1 macrophage-secreted IRF5 is positively correlated with the severity of OA symptoms.	[19,58]
		Pro-resolving lipid mediator	Synovial macrophages	Obesity-induced osteoarthritis	NA	Reduce the number of M1 macrophages and increase the formation of M2 macrophages	Preclinical stage/NA	CL attenuates obesity-induced OA by regulating the polarization of macrophages.	[59]
		Lumican (LUM)	Human primary chondrocytes and macrophages	NA	NF-κB	LUM+LPS induces the differentiation of macrophages into M1 type	Preclinical stage/NA	In the TLR4-mediated OA model, the expression of LUM is up-regulated, which promotes the formation of M1 macrophages and aggravates OA symptoms.	[23,61,62,63,64]
		Bone morphogenetic protein 7 (BMP-7)	Primary human monocytes	Patients with osteoarthritis	NA	NA	Preclinical stage/NA	BMP-7 regulates the inflammatory state of joints by changing the phenotype of OA synovium (polarizing monocytes into M2 type).	[16]
		Squid type II collagen (SCII)	RAW264.7 cells; mouse chondrocytes	Mouse OA model induced by anterior cruciate ligament transection (ACLT) and partial meniscus resection (pMMx)	STAT6	Promote the conversion of M1 macrophages into M2 macrophages	Preclinical stage/NA	SCII improves the symptoms of OA by promoting the polarization of M2 macrophages.	[68]
		Modified ZIF-8 nanoparticles	RAW264.7 cells	A mouse model of OA with anterior cruciate ligament transection (ACLT).	MAPK NF-κB JNK	Promote the transformation of M1 macrophages into M2 macrophages	Preclinical stage/NA	Modified ZIF-8 nanoparticles transform macrophages from M0 to M2 for OA treatment.	[73]

NA: none available.
